# HOSPITALIZATIONS FOR CHOLECYSTITIS AND CHOLELITHIASIS IN THE STATE OF RIO GRANDE DO SUL, BRAZIL

**DOI:** 10.1590/0102-6720201600020003

**Published:** 2016

**Authors:** Emeline Caldana NUNES, Roger dos Santos ROSA, Ronaldo BORDIN

**Affiliations:** Department of Social Medicine, School of Medicine, Federal University of Rio Grande do Sul, Porto Alegre, RS, Brazil.

**Keywords:** Cholecystitis, Cholelithiasis, Cholecystectomy, Health economics, Health management

## Abstract

**Background:**

The cholelithiasis is disease of surgical resolution with about 60,000 hospitalizations per year in the Sistema Único de Saúde (SUS - Brazilian National Health System) of the Rio Grande do Sul state.

**Aim:**

To describe the profile of hospitalizations for cholecystitis and cholelithiasis performed by the SUS of Rio Grande do Sul state, 2011-2013.

**Methods:**

Hospital Information System data from the National Health System through morbidity list for cholelithiasis and cholecystitis (ICD-10 K80-K81). Variables studied were sex, age, number of hospitalizations and approved Hospitalization Authorizations (AIH), total amount and value of hospital services generated, days and average length of stay, mortality, mortality and case fatality ratio, from health regions of the Rio Grande do Sul.

**Results:**

During 2011-2013 there were 60,517 hospitalizations for cholecystitis and cholelithiasis, representing 18.86 hospitalizations per 10,000 inhabitants/year, most often in the age group from 60 to 69 years (41.34 admissions per 10,000 inhabitants/year) and female (27.72 hospitalizations per 10,000 inhabitants/year). The fatality rate presented an inverse characteristic: 13.52 deaths per 1,000 admissions/year for males, compared with 7.12 deaths per 1,000 admissions/year in females. The state had an average total amount spent and value of hospital services of R$ 16,244,050.60 and R$ 10,890,461.31, respectively. The health region "Capital/Gravataí Valley" exhibit the highest total expenditure and hospital services, and the largest number of deaths, and average length of stay.

**Conclusion:**

The hospitalization and lethality coefficients, the deaths, the length of stay and spending related to admissions increased from 50 years old. Females had a higher frequency and higher values ​​spent on hospitalization, while the male higher coefficient of mortality and mean hospital stay.

## RESUMO

**Racional:**

A colelitíase é doença de resolução cirúrgica com cerca de 60.000 internações por ano no Sistema Único de Saúde no estado do Rio Grande do Sul.

**Objetivo:**

Descrever o perfil das internações por colecistite e colelitíase na rede pública do estado no triênio 2011-2013.

**Métodos:**

Emprego de dados do Sistema de Informações Hospitalares do Sistema Único de Saúde, através da lista de morbidades para colelitíase e colecistite (CID-10 K80-K81). As variáveis estudadas foram: sexo, idade, número de internações e de Autorizações de Internação Hospitalar aprovadas, valor total e valor dos serviços hospitalares gerados, dias e média de permanência, óbitos, coeficiente de mortalidade e letalidade, a partir das regiões de saúde do estado.

**Resultados:**

No triênio ocorreram 60.517 internações por colecistite e colelitíase, o que representou 18,86 internações por 10.000 habitantes/ano, mais frequente na faixa etária dos 60 aos 69 anos (41,34 internações por 10.000 habitantes/ano) e no sexo feminino (27,72 hospitalizações por 10.000 habitantes/ano). O coeficiente de letalidade apresentou característica inversa: 13,52 óbitos para 1.000 internações/ano para o sexo masculino, contra 7,12 óbitos para 1.000 internações/ano no sexo feminino. O estado apresentou médias de valor total gasto e de valor dos serviços hospitalares de R$16.244.050,60 e R$10.890.461,31, respectivamente. A região de saúde "Capital/Vale do Gravataí" apresentou o maior valor total gasto e de serviços hospitalares, e o maior número de óbitos, média e dias de permanência.

**Conclusão:**

Os coeficientes de internação e de letalidade, os óbitos, os dias de permanência e os gastos referentes às internações aumentam a partir dos 50 anos de idade. O sexo feminino apresentou maior frequência e maiores valores gastos com a internação, enquanto o masculino maior coeficiente de letalidade e média de permanência hospitalar.

## INTRODUCTION

The acute cholecystitis is an inflammation of the gallbladder wall. In 95% of cases occur as a result of gallstones and 5% follows other less common causes, called acute non-lithiasic cholecystitis^1^ ^,^ ^9^. Brazil has a prevalence of 9.3% of cases of cholelithiasis in the general population, requiring approximately 60,000 hospitalizations per year in the the Brazilian National Health System (SUS).

The diagnosis of cholelithiasis is performed by abdominal ultrasound with a 95% success rate and with the advantages of not being invasive examination, well tolerated by the patient and easy to perform; so, it should be the first examination performed on clinical suspicion^20^.

The treatment of acute cholecystitis involves urgent surgery. Recent meta-analysis indicates the safety and feasibility of early laparoscopic cholecystectomy till one week after onset of symptoms. Early laparoscopic cholecystectomy is considered safe in uncomplicated acute gallbladder, with low mortality (seven deaths per 10,000 operated) and lower hospital stay, less painful, with faster recovery, earlier return to work and with minor complications^17^.

The main risk factors for it are: age over 50 years, gender (females have greater risk of developing gallstones because the number of pregnancies, use of oral contraceptives and natural hormonal factors), obesity (which favors the formation of gallstones) and diabetes mellitus type 2^11^ ^,^ ^13^ ^,^ ^19^.

The incidence of gallstones - one of the main causes of morbidity in the world - should increase in coming years due to obesity and increased life expectancy, known risk factors in the development of cholelithiasis^5^. In this context, the aim of this study is to describe the profile of hospitalizations for cholecystitis and cholelithiasis in public health care for the Rio Grande do Sul, Brazil, during the triennium 2011-2013.

## METHODS

This is a population-based study, observational and cross-sectional, using data present in the Hospital Information System (SIH/SUS) through the list of morbidities in the International Classification of Diseases - 10^th^ edition (ICD 10) for gallstones and cholecystitis - codes K80 and K81, respectively.

The variables studied were gender, age, number of hospitalizations, the total amount and value of reimbursed hospital services, days and average length of stay, mortality, case fatality ratio and mortality rate from Health Regions of Rio Grande do Sul. The values of the hospitalization expenses were not update according to inflation.

Data from the Brazilian Institute of Geography and Statistics for the Census 2010 were used for the total population, by gender and age of Rio Grande do Sul. The period defined for study was the triennium 2011-2013.

For data conference were used the TabNet and TabWin applications, made available by the Brazilian Ministry of Health. The data were organized in a spreadsheet and used descriptive statistics (frequency and average). The frequency according to gender, age and Health Region was expressed by the number of admissions divided by the population, multiplied by 10,000 per year. The coefficient of mortality was calculated by dividing the total number of deaths for each indicator by the number of hospital admissions related to age, gender and Health Region, and the result was multiplied by 1,000 admissions per year. The mortality rate was calculated by dividing the total number of deaths related to age, gender and Health Region by the population, and 100,000 per year multiplied the result.

By having used public access database, there was no need for referral to the Ethics Committee in Research.

## RESULTS

In the period of 2011 to 2013 were paid 60,517 hospitalizations for cholecystitis and cholelithiasis in the public health system with an annual average of 20,172 hospitalizations, representing 18.86 hospitalizations per 10,000 population/year.

The systematization by gender (Table 1) pointed that the women showed the largest number of hospitalizations, total amount spent, amount of hospital services, days of stay, number of deaths and mortality rate. On the other hand, the average length of stay and mortality coefficient was higher among men.

**TABLE 1:** Indicators related to gender - cholecystitis and cholelithiasis

Indicators (average of triennium)	Admissions (per 10.000 population/year)	Lethality coefficient (per 1.000 admissions/year)	Total spent value (annual average) (R$)	Hospitalar services cost (R$)	Mean cost for hospitalization (R$)	Days of stay	Average stay time (days)	Nº of deaths	Mortality coefficient (per 100.000 hab/year)
Gender									
Males	9.53	13.52	4.093.544,53	2.865.771,87	820,65	24.145	5	67	1,29
Females	27.72	7.12	12.150.506,07	8.024.689,45	792,27	56.042	4	109	1,99
Total	18.86	8.70	16.244.050,60	10.890.461,31	799,23	80.188	4	176	1,65

In Table 2 are the indicators by age. The age group of 0-4 years old had the highest average number of days of stay and average amount reimbursed per hospitalization; the 50-59 concentrated the highest annual average expenditure values, value of hospital services and stay days. Although the range of 60-69 years old has shown the highest rate of hospitalizations (41.34 per 10,000 population/year) the range of 80 years old and above experienced the highest number of deaths (48), mortality rate (23.77 deaths/100.000 people/year) and mortality (80 deaths/1.000 admissions/year).

**TABLE 2:** Indicators related to age - cholecystitis and cholelithiasis

Indicators (average of triennium)	Admissions (per 10.000 people/year)	Lethality (per 1.000 admissions/year)	Total spent value (annual average) (R$)	Hospitalar services cost (R$)	Mean cost for hospitalization (R$)	Days of stay	Average stay time (days)	Nº of deaths	Mortality (per 100.000 people/year)
Age (years)									
0 - 4	0,25	0	9.321,68	7.723,93	1.210,19	67	9	0	0
5 - 9	0,27	0	14.147,89	10.854,96	709,80	68	3	0	0
10 - 14	1,10	0	74.518,42	54.419,22	783,37	393	4	0	0
15 - 19	4,75	0	320.484,02	215.011,44	762,69	1.543	4	0	0
20 -a 29	11,42	1,49	1.551.508,35	1.019.815,74	762,38	7.178	4	3	0,17
30 - 39	21,04	1,13	2.539.792,07	1.647.967,55	771,27	10.698	3	4	0,24
40 - 49	27,22	3,68	3.290.239,40	2.149.746,17	784,66	14.306	3	15	1,00
50 - 59	35,81	4,14	3.681.574,96	2.413.020,75	796,03	17.057	4	19	1,49
60 - 69	41,34	12,35	2.771.340,59	1.884.808,78	822,85	14.692	4	41	5,7
70 - 79	36,77	27,59	1.467.016,85	1.071.062,37	881,98	9.743	6	46	10,20
80 years and over	29,26	80,01	514.784,69	408.306,46	865,64	4.376	7	48	23,77
Total	18,86	8,70	16.244.050,60	10.890.461,31	799,23	80.188	4	176	1,65

The state of Rio Grande do Sul is divided into 30 Health Regions^16^ and into 19 administrative regions of the State Health Department^15^.Table 3 shows the indicators studied by Health Region.

**TABLE 3:** Indicators related to Health Region - cholecystitis and cholelithiasis

Indicators (average of triennium)	Admissions (per 10.000 hab/year)	Lethality coefficient (per 1.000 admissions/year)	Total spent value (annual average) (R$)	Hospitalar servicescost (R$)	Mean cost for hospitalization (R$)	Days of stay	Average stay time (days)	Nº of deaths	Mortality coefficient (per 100.000 hab/year)
Health Regions									
Verdes Campos	22,73	4,24	674.225,08	480.852,30	703,33	2.926	3	4	0,96
Entre Rios	16,53	7,80	142.065,98	83.371,64	678,64	668	3	2	1,34
FronteiraOeste	23,80	11,37	786.913,94	544.455,75	706,80	3.927	4	13	2,72
BelasPraias	25,71	6,37	364.875,42	168.028,73	1.007,59	1.009	3	2	1,69
BonsVentos	14,50	9,57	223.715,09	127.164,45	743,43	901	3	3	1,31
V.Paranhana/C. Serra	17,75	8,91	267.836,05	171.790,86	698,61	1.415	4	3	1,57
Vale dos Sinos	17,27	10,18	991.659,57	636.685,93	745,60	4.741	4	13	1,76
Vale Caí/Metropolitan	18,59	9,15	1.064.315,82	728.592,59	791,14	4.218	3	13	1,76
Carbonífera/Costa Doce	8,51	5,19	237.441,25	163.052,43	703,04	971	3	2	0,52
Capital/Vale Gravataí	19,17	7,17	3.594.347,63	2.736.333,18	841,76	23.865	6	31	1,38
SetePovosdas Missões	23,58	9,11	545.335,54	347.947,18	796,66	2.171	3	6	2,10
Portal das Missões	17,75	12,04	196.830,91	128.775,99	728,24	1.335	5	3	1,97
Diversidade	26,66	9,61	459.767,84	303.361,90	775,75	1.921	3	6	2,54
FronteiraNoroeste	18,30	10,05	328.582,51	215.036,53	781,92	1.146	3	4	1,91
Caminho das Águas	23,11	7,40	408.322,33	202.341,53	908,63	1.165	3	3	1,78
Alto UruguaiGaúcho	17,43	9,14	367.056,48	235.094,65	913,68	1.193	3	4	1,59
Planalto	32,75	8,51	1.269.305,57	781.528,46	989,24	4.868	4	11	2,79
Araucárias	13,17	0,00	133.995,44	72.222,70	765,46	521	3	0	0,00
Botucaraí	17,01	13,31	144.759,91	85.190,54	731,16	590	3	2	2,02
Rota da Produção	13,31	9,51	140.887,84	85.667,47	658,03	757	4	2	1,24
Sul	19,67	7,17	1.354.819,27	874.361,71	810,37	7.094	4	12	1,42
Pampa	23,55	18,58	393.294,51	239.680,78	903,39	1.926	4	8	4,38
Caxias e Hortênsias	12,73	12,01	576.846,81	405.399,44	846,92	3.173	5	8	1,50
Campos Cima da Serra	20,95	13,36	113.792,47	83.116,09	571,59	754	4	3	2,81
Vinhedos e Basalto	14,86	10,81	308.582,51	213.093,18	736,90	1.368	3	4	1,55
Uva Vale	14,17	1,24	167.841,68	109.474,07	691,02	719	3	0	0,20
Jacuí Centro	19,85	12,58	262.446,94	183.371,97	659,96	1.489	4	5	2,50
Vinte e Oito	15,21	9,98	355.247,68	245.141,15	712,53	1.661	3	5	1,53
Vales e Montanhas	9,36	9,60	118.333,76	79.823,00	613,35	735	4	2	0,80
Vale da Luz	26,76	9,56	250.604,76	159.505,12	768,99	962	3	3	2,27
Total	18,86	8,70	16.244.050,60	10.890.461,31	799,23	80.188	4	176	1,65

As would be expected, by concentrating the highest population quantitative and installed technology base, the Health Region "Capital/Vale do Gravataí" (including Porto Alegre, the state capital) had the highest total amount spent and value of hospital services, of days and average length of stay, and number of deaths.

Health region "Planalto" presented the highest rate of hospitalizations (32.75/ 10,000 people/year) and the region of the "Pampa" the highest mortality rates (4.38/100,00 people/year) and mortality (18,58 deaths/1,000 admissions/year), all above indicators to the region "Capital/Vale do Gravataí."

## DISCUSSION

Higher prevalence of hospital admissions was observed in the female group (major quantities regarding the occurrence of hospitalizations, total amount spent, amount of hospital services, days of stay, number of deaths and mortality rate), while the average stay and the coefficient of mortality were higher in men, looking to demonstrate disease with different natural evolution in both gender. Hypotheses for the difference in gravity for the lithiasic disease would be derived from the anthropometric characteristics, body fat distribution and pain threshold^13^.

Analysis by age shows that there was an increase of the occurrence with the increase of age, culminating in the population corresponding to the age group from 60-69 years old (n=808,630)^3^, which showed the highest rate of hospitalization for cholecystitis and cholelithiasis (41.34 admissions per 10,000 people/year), confirming others studies^5^ ^,^ ^19^.

Evidence in uncomplicated acute gallbladder disease suggests that early laparoscopic cholecystectomy is safe and reduces the period of hospitalization^18^. Mean surgical time for laparotomic and laparoscopic cholecystectomies are generally similar between the elderly and younger, and the hospital stay time is higher in elderly patients undergoing laparotomy^12^.

Triennium greatest average of hospital stay time occurred in children under four years of age, followed by those with 70 years or more. A longer hospital stay in elderly patients is usually related to more complications, as they require special care in their preparation and postoperative care and multiprofessional care^12^.

There were no deaths in the age group 0-19 years old, but the case fatality ratio started to grow with increasing age from 30 years old, culminating in the incidence of 80.01 deaths per 1,000 admissions/year in the 80 years or older group.

Usually, biliary calculi disease is asymptomatic, and a very important aspect is clinical manifestation, with frequent acute exacerbation and complicated forms of the disease, increasing from 3 to 7 times the mortality in the emergency procedure, when compared to the elective^14^. This study does not provide data if the procedure was performed on an emergency basis or elective; so, it was not possible to confirm whether or not this situation.

The total amount spent represents the amount related to bills paid, being higher in the age group of 50-59 years old accounted for 22.6% of the average annual spending in three years. This same age group also presented the highest annual average for the value of hospital services generated for hospitalizations for cholecystitis and cholelithiasis. The highest average value of admissions for cholecystitis and cholelithiasis occurred among children under four years of age, with an average of R$ 1,210.19, higher than 51% to the total average.

The total days of hospitalization, counted between hospital admission and discharge (day stay)^4^ was higher among 50-59 years old (21.3% of total). The highest average stay occurred in the age group of 0-4 years old (nine days), followed by those aged 80 or more (seven days), above the state average of four days of stay.

The number of deaths increased proportionally with age, not being registered any among children under 19. The age above 80 had 48 (27%) of the 176 deaths. The fact resulted in higher mortality rate (23.77 per 100,000 population/year), much higher than that found for the state in the triennium 2011-2013 (1.65 per 100,000 population/ year).

The prevalence of gallstones varies according to the continent, country, state and city. It can vary, even according to the patient groups^8^. The total population of the state is 10,693,929 inhabitants^3^, but the distribution of hospitalizations and indicators in a study by Health Region showed geographic heterogeneity. It is noteworthy that the Health Region "Planalto" with 382,429 inhabitants, presented higher admissions coefficient compared to other regions, with an average of 32.75 admissions/10,000 population/year. The Health Region "Pampa" got the highest mortality rates (4.38/100,000 population/year) and mortality (18.58 deaths/1000 admissions/year). The Health Region "Capital/Vale do Gravataí" with 2,225,237 inhabitants and incorporating the state capital, Porto Alegre, presented 19.17 admissions/10,000 population/year. And as expected for having the largest population size and installed services, had the highest total amount spent and value of hospital services, days and average length of stay, and number of deaths (Table 3). Finally, the Health Region "Campos de Cima da Serra" had the lowest average cost of hospitalization for cholecystitis and cholelithiasis, with R$ 571.59 and the lowest total expenditure.

## CONCLUSION

From 50 years old increases the frequency and mortality rates, deaths, days of stay and expenses related to hospitalization. Females had higher frequency and higher expenses with the hospital, while the male had higher coefficient of lethality and average hospital stay. The Health Region "Capital/Vale Gravataí", the most populous, had the highest total amount spent and value of hospital services, days and average length of stay, and number of deaths. Modifiable risk factors (overweight, diabetes mellitus type 2) can reduce the occurrence of cholelithiasis, associated with preventive health programs helping to reduce complications inherent to this disease.
